# P-1402. Will You Receive HIV and HCV Testing from a Mobile Pharmacy? Pre-implementation Needs Assessment and Pilot Findings from the United States First Legal Retail Mobile Pharmacy

**DOI:** 10.1093/ofid/ofae631.1577

**Published:** 2025-01-29

**Authors:** Adati Tarfa, Cynthia Frank, Ralph P Brooks, Alysse Schultheis, Angela Di Paola, Sheela Shenoi, Sandra Ann Springer

**Affiliations:** Yale University, New Haven, Connecticut; Yale School of Medicine, New Haven, Connecticut; Yale School of Medicine - AIDS Program, New Haven, Connecticut; Yale School of Medicine, New Haven, Connecticut; Yale University, New Haven, Connecticut; Yale University, New Haven, Connecticut; Yale University, New Haven, Connecticut

## Abstract

**Background:**

Connecticut enacted Senate Bill No.1102 in July 2023, allowing mobile pharmacies to operate in the state to meet the needs of individuals at risk for and living with HIV, including people who use drugs (PWUD), who often experience difficulties accessing and navigating health care systems. The mobile pharmacy and clinic (MPC) care delivery model uses clinicians, community health workers (CHW), and pharmacists to provide healthcare to people where they live (housed/unhoused), improving accessibility to care for people living with/at risk of HIV as well as Hepatitis C (HCV) and substance use disorder (SUD) treatment. We present pre-implementation needs assessment and pilot data of an MPC from an urban area of Connecticut.

**Figure:**
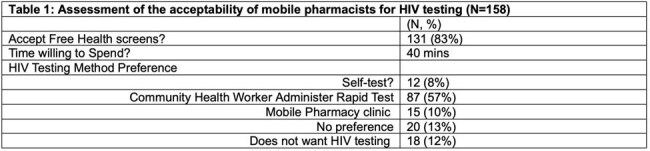

**Methods:**

CHWs conducted a needs assessment of community members from 08/2023 to 11/2023 to characterize barriers to accessing care and the acceptability of health screenings, including rapid HIV and HCV testing. Pilot implementation data includes service use, clinical assessment, and prescription fill data of the MCP from 12/2023 to 04/2024.

**Figure:**
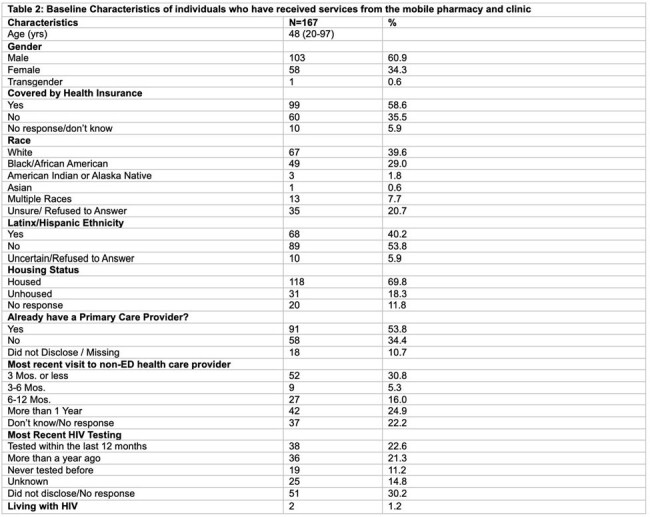

**Results:**

Needs assessment: Among 158 respondents, the main barriers to accessing pharmacy services were transportation (67%) and medication cost (24%). The majority (89%) showed willingness to use a mobile pharmacy, and 57% were willing to receive HIV testing by a CHW (Table 1). Implementation: Among 167 individuals who received services from the MPC, a majority were male (62%), White (40%), and ethnicity of Latinx/Hispanic (42%), 11% had never tested for HIV, and 2 were living with HIV (Table 2). Among 38% accepting HIV testing, none tested positive and were all offered PrEP. Also, 56% accepted HCV testing, 10 (20%) had a positive viral load, and 8 (80%) started treatment. Of those screened, 35% (20/57) and 18% (12/66) were positive for Alcohol Use disorder (AUD) and Opioid Use Disorder (OUD), respectively. Of the 284 medications dispensed,10 (4%) were for PrEP, 2 (1%) for long-acting injectable ART, 9 (3%) for AUD, and 4 (1%) for OUD (Table 3).

**Figure:**
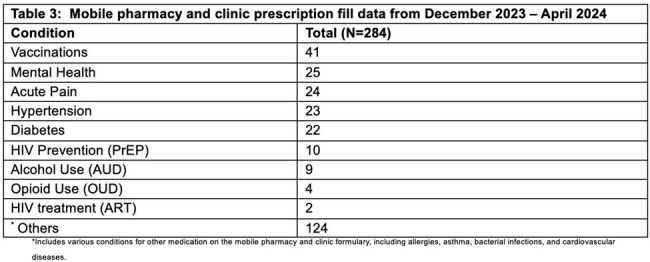

**Conclusion:**

The MPC can respond to the needs of an urban population when current services are impractical for patients. Providing health services through an MPC is feasible and should be expanded to meet gaps in health care.

**Disclosures:**

**Ralph P. Brooks, MS**, Merck: Stocks/Bonds (Public Company) **Sheela Shenoi, MD MPH**, Merck Pharmaceuticals: Stocks/Bonds (Private Company) **Sandra Ann Springer, MD**, Alkermes Inc: Honoraria|Alkermes Inc: In kind study drug donation for NIH sponsored research|Indivior Pharmaceutical company: In kind study drug donation for NIH sponsored research

